# Regulation of *gastrin-releasing peptide* gene expression in chickens

**DOI:** 10.3389/fphys.2026.1940305

**Published:** 2026-10-21

**Authors:** Kefan Tang, Xuan Tian, Xiaohan Yuan, Yanrong Li, Jiannan Zhang, Yajun Wang, Juan Li

**Affiliations:** 1 Key Laboratory of Bio-resources and Eco-environment, Ministry of Education, College of Life Sciences, Sichuan University, Chengdu, China; 2 Animal Disease Prevention and Green Development Key Laboratory of Sichuan Province, Chengdu, China

**Keywords:** chicken, gastrin-releasing peptide, gene expression, pituitary, sex steroids

## Abstract

Gastrin-releasing peptide (GRP) is a neuropeptide with diverse physiological functions in mammals. Similar to its mammalian counterparts, chicken GRP (cGRP) is expressed in endocrine cells of the gastrointestinal tract, including the proventriculus, where it regulates gastric acid secretion. Our previous study showed that cGRP is predominantly expressed in the anterior pituitary and acts as a novel pituitary hormone that promotes the survival of small ovarian follicles. Using primary pituitary cell cultures, we found that gonadotropin-releasing hormone 1 (GnRH1) markedly increased *cGRP* mRNA abundance in both dose- and time-dependent manners. Pharmacological analyses suggested that this effect likely involved the AC/cAMP/PKA pathway and the PLC/PKC/MEK/ERK pathway. We also examined the regulation of *GRP* expression by sex steroids. 5α-dihydrotestosterone (DHT) and progesterone (P4) downregulated *GRP* expression in cultured chicken pituitary cells, whereas 17β-estradiol (E2) upregulated *GRP* expression in dose- and time-dependent manners. Analysis of the isolated *GRP* 5′-flanking region identified potential estrogen receptor-binding sites, providing a molecular basis for E2-mediated upregulation of *GRP* expression. These findings support cGRP as a novel pituitary hormone under hypothalamic GnRH1 control and feedback regulation by sex steroids. Together with its reported role in the survival of small ovarian follicles, cGRP may contribute to the regulation of egg production in chickens.

## Introduction

Gastrin-releasing peptide (GRP) is a member of the bombesin family that regulates diverse physiological processes. For example, GRP participates in appetite regulation in rats and mice (Roesler and Schwartsmann, 2012; Zhao et al., 2023) and in itch signaling in mice and humans (Sun and Chen, 2007; Bourane et al., 2015; Misery et al., 2023). In male mice, spinal GRP neurons contribute to sexual function (Sakamoto et al., 2008; Tamura et al., 2017). In mammals, GRP also stimulates intestinal motility and gastric acid secretion (Jensen et al., 2008). By binding to its cognate receptor, GRP receptor (GRPR), GRP activates downstream MAPK/ERK signaling. This mitogenic activity has been implicated in the development of several tumors (Cuttitta et al., 1985; Faviana et al., 2021; Kunos et al., 2023).

In chickens, GRP is abundantly expressed in endocrine cells of the gastrointestinal tract, including the proventriculus (Buffa et al., 1982; Yamada et al., 1985; Dubbeldam and den Boer-Visser, 2000), and contributes to gastric acid secretion and pancreatic enzyme release (Campbell et al., 1991, 1994; Tachibana et al., 2010). Studies by Cline’s group further suggest that cGRP participates in the regulation of feeding and satiety (Tachibana et al., 2010; Bohler et al., 2019). The subsequent research showed that cGRP is also abundantly expressed in the chicken pituitary (Mo et al., 2017). Single-cell RNA-seq data also detected *GRP* expression in chicken ovarian tissue (Leng et al., 2024). Because cGRPR is abundant in granulosa cells of small ovarian follicles, GRP may act as a mitogen that promotes the survival of these follicles (Mo et al., 2025).

The abundance of cGRP mRNA and protein in the caudal and cephalic lobes of the chicken pituitary (Mo et al., 2017), raised the question of whether this putative pituitary hormone is regulated by hypothalamic factors. GRP-immunoreactive cells primarily co-localize with luteinizing hormone (LH)-immunoreactive cells (Mo et al., 2017), suggesting that GRP may be regulated by gonadotropin-releasing hormone (GnRH), the central regulator of reproductive hormone synthesis and secretion. Moreover, pituitary hormone expression is often feedback regulated by sex steroids (Scanes, 2022a). The effects of sex steroids on *cGRP* expression remain unclear. In this study, we investigated the regulation of *cGRP* expression by GnRH1 and sex steroids. Because 17β-estradiol (E2) increased *cGRP* expression, we further isolated the 5′-flanking region of the *GRP* gene and identified potential estrogen receptor-binding sites. Together with the reported mitogenic effect of GRP on granulosa cells for the survival of small ovarian follicles (Mo et al., 2025), these findings support the hypothesis that GRP plays a role in the regulation of egg production in chickens.

## Materials and methods

### Ethics statement

All animal experiments were conducted in accordance with the guidelines for experimental animals issued by the Ministry of Science and Technology of the People’s Republic of China. All animal experimental protocols used in this study were approved by the Animal Ethics Committee of College of Life Sciences, Sichuan University (SCU230303012). Lohmann layer chickens purchased from MUXING Company (Chengdu, Sichuan, China) were used in this study.

### Chemicals, hormones, antibodies, and primers

Chicken GnRH1 was synthesized using solid-phase Fmoc chemistry (GL Biochem, Shanghai, China). The pharmacological agents forskolin, MDL-12,330A, H89, phorbol 12-myristate 13-acetate (PMA), calphostin C (Cal-C), PD98059 (PD), and ionomycin were purchased from Calbiochem (Merck KGaA, Darmstadt, Germany). The sex steroids including 5α-dihydrotestosterone (DHT),17β-estradiol(E2), progesterone (P4) and diethylstilbestrol (DES) were also purchased from Calbiochem (Merck KGaA, Darmstadt, Germany). All primers were synthesized by Beijing Genomics Institute (BGI, China) and are listed in **Table 1**.

**Table 1:** Primers used in this study.

Gene	Sense/antisense	Primer sequence (5’- to- 3’)	Size (bp)
Primers for quantitative real-time PCR assay			
*GRP*	Sense Antisense	CCCGCGCTCACCAAGATCTA GGTGATGCTGACAATGGGATCT	128
*β-actin*	Sense Antisense	CCCAGACATCAGGGTGTGATG GTTGGTGACAATACCGTGTTCAAT	123
*FSHβ*	Sense Antiense	GTGGTGCTCAGGATACTGCT CAGTGGCACTCTGTAGCTAC	174
*GAPDH*	SenceAntisence	TGCTGCCCAGAACATCATCCATCAGCAGCAGCCTTCACTACC	199
Primers for PCR assay			
*p(-1914/+86)Luc*	Sense Antisense	5’-ATCCTTTCACCTGTGGGATGGACTCACG-3’ 5’-GCCGTCGAGTCGCACACCGC-3’	2000
*p(-914/+86)Luc*	Sense Antisense	5’-AAAGTCACGTTGTCCTTTGAGTTTGCTCATTTTCC-3’ 5’-GCCGTCGAGTCGCACACCGC-3’	1000
*p(-414/+86)Luc*	Sense Antisense	5’-TAATGGTCAAAACGGCCCCGAGC-3’ 5’-GCCGTCGAGTCGCACACCGC-3’	500
*p(-164/+86)Luc* *ESR1* *ESR2* *p(-1914/+86)mut1 Luc*	Sense Antisense Sense Antisense Sense Antisense Sense Antisense	5’-GGGTCCGGGCTGCCCGCGGT-3’ 5’-GCCGTCGAGTCGCACACCGC-3’ 5’-ATGACCATGACCCTTCACACC-3’ 5’-GGTGGCTAATGTCATTGGAGTG-3’ 5’-ATGTCCCTCTGTGCATCTTCTC-3’ 5’-TCAGACCTGGAAATGTGAAACTTG-3’ 5’-CTCGAAGCTTATTGAGTTTGCTCATTTTCCAGTG-3’ 5’-AGCAAACTCAATAAGCTTCGAGGAAAAGAGTTTC-3’	250 1883 1626 6807
*p(-1914/+86)mut2 Luc*	Sense Antisense	5’-CACAGCTGGCCGGAGTGCTGGAGCACTGCAAG-3’ 5’-TCCAGCACTCCGGCCAGCTGTGTTAATGAGTGGA-3’	6807

### Pituitary cell culture and RNA extraction

The anterior pituitaries were collected from one-week-old chicks. In the present study, due to the limited number of pituitary cells available from each chick, anterior pituitaries were collected from a number of one-week-old chicks which constitute of male and female at a 1:1 ratio in every sample collection. As described previously (Liu et al., 2022), the collected anterior pituitaries were further divided into different groups based on the differential experimental requirements. The anterior pituitaries in each group were then sliced and digested with 0.25% trypsin at 37 °C for 20 min. Dispersed pituitary cells were cultured at a density of (1-5) ×10^5^ cells/well in Medium 199 supplemented with 15% fetal bovine serum in Corning CellBIND 48-well plates (Corning, Tewksbury, MA, USA) at 37 °C under 5% CO_2_.

For the effects of chicken GnRH1 on chicken *GRP* mRNA expression study and the receptor mediated signaling pathway revelation, the dispersed cells were cultured for 24 h, and then their medium were replaced with serum-free Medium 199. The cells (300 μL/well) were further treated with chicken GnRH1 for 12–48 h or with increasing concentrations (0.1–100 nM) in the presence or absence of pharmacological agents.

For the effects of DHT, P4 and E2 on *GRP* expression study, the dispersed cells were cultured for 24 h, and then the medium were replaced with serum-free medium 199. The cells (300 μL/well) were further treated with DHT, P4, E2 or DES for 12–48 h or with various concentrations (0.1–100 nM) of the compounds of interest.

For the differential regulation of *GRP* and *FSHβ* expression by E2 in cultured pituitary Cells, the dispersed cells were cultured for 24 h, and then their medium were replaced with serum-free Medium 199. The cells (300 μL/well) were further treated with E2 (20 nM, 24h).

To extract total RNA from cultured cells, the medium was first removed from each plate. Tri Reagent (300 μL; Molecular Research Center, Inc.) was added to each well, and the plate was shaken for 10 min at 600 rpm using a Thermomixer Comfort (Eppendorf, Hamburg, Germany). The lysate from each well was transferred to a microtube containing 75 μL chloroform, vortexed, and centrifuged for 30 min at 4 °C. The aqueous phase was transferred to a new tube containing isopropanol, vortexed for 30 s, incubated at -20 °C for 30 min, and centrifuged for 30 min at 4 °C. After washing with 75% ethanol and brief air-drying, the RNA pellet was dissolved in 10 μL DEPC-treated H_2_O and stored at -80 °C. RNA concentration and purity were determined using a One Drop 1000 spectrophotometer, as described previously (Wan et al., 2023).

### Quantitative real-time polymerase chain reaction

For reverse transcription, total RNA (2 μg) and oligo(dT) (0.5 μg) were mixed in a final volume of 5 μL, incubated at 70 °C for 10 min, and cooled at 4 °C for 2 min. First-strand buffer, each deoxynucleotide triphosphate (dNTP; 0.5 mM), and 100 U Moloney murine leukemia virus (MMLV) reverse transcriptase (Takara) were then added to a final reaction volume of 10 μL. Reverse transcription was performed at 42 °C for 90 min. The resulting cDNA was diluted with Milli-Q water and stored at -20 °C, as described previously (Zhu et al., 2021).

Chicken *GRP* mRNA abundance was measured by quantitative real-time PCR (qPCR) using our previously established method (Jiang et al., 2022). Primers for chicken *GRP* (GenBank accession no. NM_001277900; NCBI Gene ID: 425213) were designed from the GenBank sequence and listed in **Table 1**. Briefly, primers (10 μM), dNTPs (10 mM), EasyTaq buffer, EasyTaq DNA polymerase (TransGen Biotech), EvaGreen (Biotium), Milli-Q water, and cDNA template were mixed in a final volume of 20 μL. Reactions were performed using a CFX96 Real-Time PCR Detection System (Bio-Rad). The amplification program comprised an initial denaturation at 94 °C for 10 min, followed by 40 cycles of denaturation at 94 °C for 20 s, annealing at 60 °C for 15 s, and extension at 72 °C for 30 s. *β-actin* and *GAPDH* were selected as the reference genes based on their stable transcript expression in chicken pituitary tissues and cells, as previously validated in our studies (Mo et al., 2017, 2025). Primer specificity was confirmed by melt-curve analysis and Sanger sequencing of the amplicons, and Ct values from technical qPCR replicates were averaged before 4 biological replicates were included in statistical analyses.

Chicken *FSHβ* mRNA abundance was measured by quantitative real-time PCR (qPCR) following the method introduced above. Primers for chicken *FSHβ* (GenBank accession no. NM_204257.2; NCBI Gene ID: 374108) were designed from the GenBank sequence and listed in **Table 1**.

### Isolation of the 5′-flanking region of the chicken GRP gene

The 5′-flanking region of the chicken *GRP* gene was cloned to investigate its transcriptional regulation profile. The full-length chicken GRP cDNA sequence was aligned with the chicken genome sequence available in Ensembl (www.ensembl.org/Gallus_gallus/). Sense and antisense primers (**Table 1**) were designed to amplify the 5′-flanking region from chicken genomic DNA. PCR was performed with an initial denaturation at 95 °C for 2 min, followed by 30 cycles of denaturation at 94 °C for 20 s, annealing at 58 °C for 10 s, and extension at 72 °C for 1 min 30 s, with a final extension at 72 °C for 7 min. The amplified products were subcloned into pBluescript SK (+/-) and subjected to sequence analysis.

### Promoter analysis of the chicken GRP gene in cultured cells

To determine whether the 5′-flanking region upstream of the *GRP* transcription start site exhibited promoter activity, a series of constructs containing progressively truncated fragments was generated by PCR. Constructs preparation and promoter analyses were performed as described previously (Li et al., 2012; Wang et al., 2024).

For the analysis of basal promoter activity, the primers used to generate each construct are listed in **Table 1**. PCR products were first cloned into pBluescript SK (+/-) for sequence confirmation and then subcloned into the pGL3-Basic vector (Promega). Chicken embryo fibroblast (DF-1) and human embryonic kidney (HEK293) cells were cultured as described previously (Wan et al., 2025). For transfection, 1 × 10^5^ cells were seeded in each well of a 48-well plate. A mixture containing 100 ng promoter-luciferase construct, 5 ng pRL-TK (Promega), and 1.5 μL Lipofectamine (Invitrogen) was prepared in 20 μL PBS. Following the manufacturer’s instructions, firefly luciferase activity was normalized to Renilla luciferase activity derived from the pRL-TK vector. Promoter activity was expressed as the fold change relative to cells transfected with the pGL3-Basic control vector. Data were analyzed by one-way ANOVA followed by Dunnett’s test using GraphPad Prism 5 (GraphPad Software, San Diego, CA, USA). All experiments were repeated at least twice.

### Effects of E2 on chicken *GRP* promoter activity and identification of potential estrogen receptor-binding sites

To examine the effects of E2 on *GRP* promoter activity, the E2 receptors expression constructs: pcDNA3.1-ESR1 and pcDNA3.1-ESR2 were generated. The primers used to generate the above constructs are listed in **Table 1**. The serial deletion constructs: p(-1914/+86)-Luc, p(-914/+86)-Luc, p(-414/+86)-Luc, and p(-164/+86) were co-transfected into DF-1 cells with pcDNA3.1-ESR1, pcDNA3.1-ESR2, and pRL-TK. A promoter-less pGL3-Basic vector (pGL3) was included as a control. At 24 h after transfection, the cells were treated with 100 nM E2. Luciferase activity was measured and normalized as described above.

To identify the potential estrogen receptor binding sites, three site-directed deletion constructs were constructed based on the p(-1914/+86)-Luc construct. The single site 1 (estrogen receptor 1 binding site, ESR1) deletion mutant: p(-1914/+86)-mut1-Luc was constructed based on the primer pairs flanking the ESR1 (**Table 1**). The single site 2 (estrogen receptor 2 binding site, ESR2) deletion mutant: p(-1914/+86)-mut2-Luc was constructed based on the primer pairs flanking the ESR2 (**Table 1**). The double sites (ESR1/ESR2) deletion mutant: p(-1914/+86)-mut1/2-Luc was constructed based on the primer pairs flanking ESR1 and ESR2 (**Table 1**). The constructs were co-transfected into DF-1 cells with pcDNA3.1-ESR1, pcDNA3.1-ESR2, and pRL-TK. The pRL-TK vector was used to normalize transfection efficiency, and a promoterless pGL3-Basic vector was included as a control. At 24 h after transfection, the cells were treated with 100 nM E2. Luciferase activity was measured and normalized as described above.

### Statistical analysis

Relative *GRP* mRNA abundance in pituitary cells or pituitary tissue was first normalized to β-actin and then expressed relative to the corresponding control value. Data are presented as mean ± SEM. For primary pituitary-cell experiments, four biological replicates were included per condition in the representative experiment shown; all experiments were independently repeated three times.

For luciferase assays, three independently transfected wells were one representative experimental unit. All the experiments were independently repeated at least twice. Two-group comparison were performed using two-tailed Student’s t-tests. One-factor and two-factor experiments were analyzed by one-way ANOVA with Dunnett’s test and two-way ANOVA with Bonferroni’s *post hoc* test, respectively. Analyses were performed using GraphPad Prism 5, and P < 0.05 was considered statistically significant.

## Results

### Localization of *GRP* in chicken pituitary gonadotrophs

Our previous study showed that *cGRP* is predominantly and abundantly expressed in the anterior pituitary (Mo et al., 2017). This high expression prompted us to examine its distribution among pituitary cell clusters based on our published adult chicken anterior pituitary scRNA-seq dataset (Zhang et al., 2021). *cGRP* was detected primarily in the gonadotroph cluster of female chickens, with additional expression in lactotrophs and folliculo-stellate cells (**Figure 1**). Analysis of the domestic chicken multi-tissue gene expression database (https://chickenatlas.avianscu.com/) (Zhang et al., 2022) further showed that *cGRP* and *cGnRHR2* mRNAs were highly abundant in the anterior pituitaries of adult chickens.

**Figure 1:**
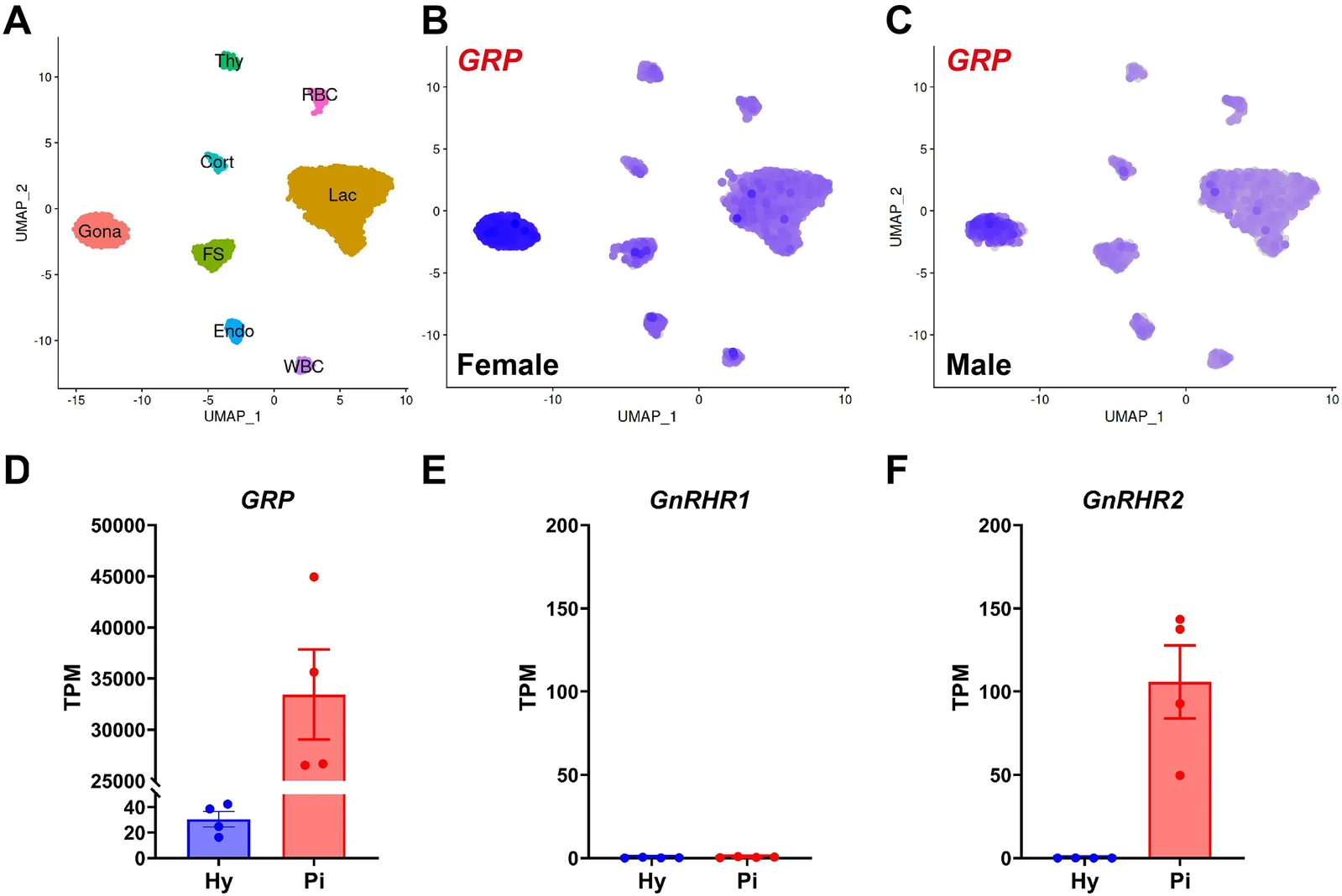
Single-cell transcriptomic analysis showing high *GRP* expression in female chicken pituitary gonadotrophs which was analyzed based on a published adult chicken anterior pituitary scRNA-seq dataset (Zhang et al., 2021). **(A)** Uniform Manifold Approximation and Projection (UMAP) plot showing eight identified pituitary cell clusters. Cells are colored according to Seurat clustering and annotated by cell type; each point represents one cell. Gona, gonadotrophs; Lac, lactotrophs; Cort, corticotrophs; Thy, thyrotrophs; FS, folliculo-stellate cells; Endo, endothelial cells; WBC, white blood cells; RBC, red blood cells. **(B)** UMAP plot showing *GRP* expression across cell clusters in the female chicken pituitary. *GRP* was broadly expressed, with the highest expression in gonadotrophs. **(C)** UMAP plot showing *GRP* expression across cell clusters in the male chicken pituitary. **(D–F)** RNA-seq analysis of *GRP*, *GnRHR1*, and *GnRHR2* mRNA abundance in the hypothalamus (Hp) and anterior pituitary (Pi) of adult chickens, based on the domestic chicken multi-tissue gene expression database established in our previous studies (https://chickenatlas.avianscu.com/).

### Regulation of *GRP* expression by GnRH1 in cultured chicken pituitary cells

The localization of *GRP* in gonadotrophs prompted us to investigate its regulation by GnRH1, a major regulator of pituitary reproductive function (Sharp et al., 1990). As shown in **Figure 2A**, 100 nM GnRH1 significantly increased *GRP* mRNA abundance in cultured chicken pituitary cells, with the strongest response observed after 6 h treatment. qPCR analysis further showed that GnRH1 (0.1–100 nM for 6 h) increased *GRP* mRNA abundance in a dose-dependent manner, with a significant response at concentrations as low as 0.1 nM (**Figure 2B**).

**Figure 2:**
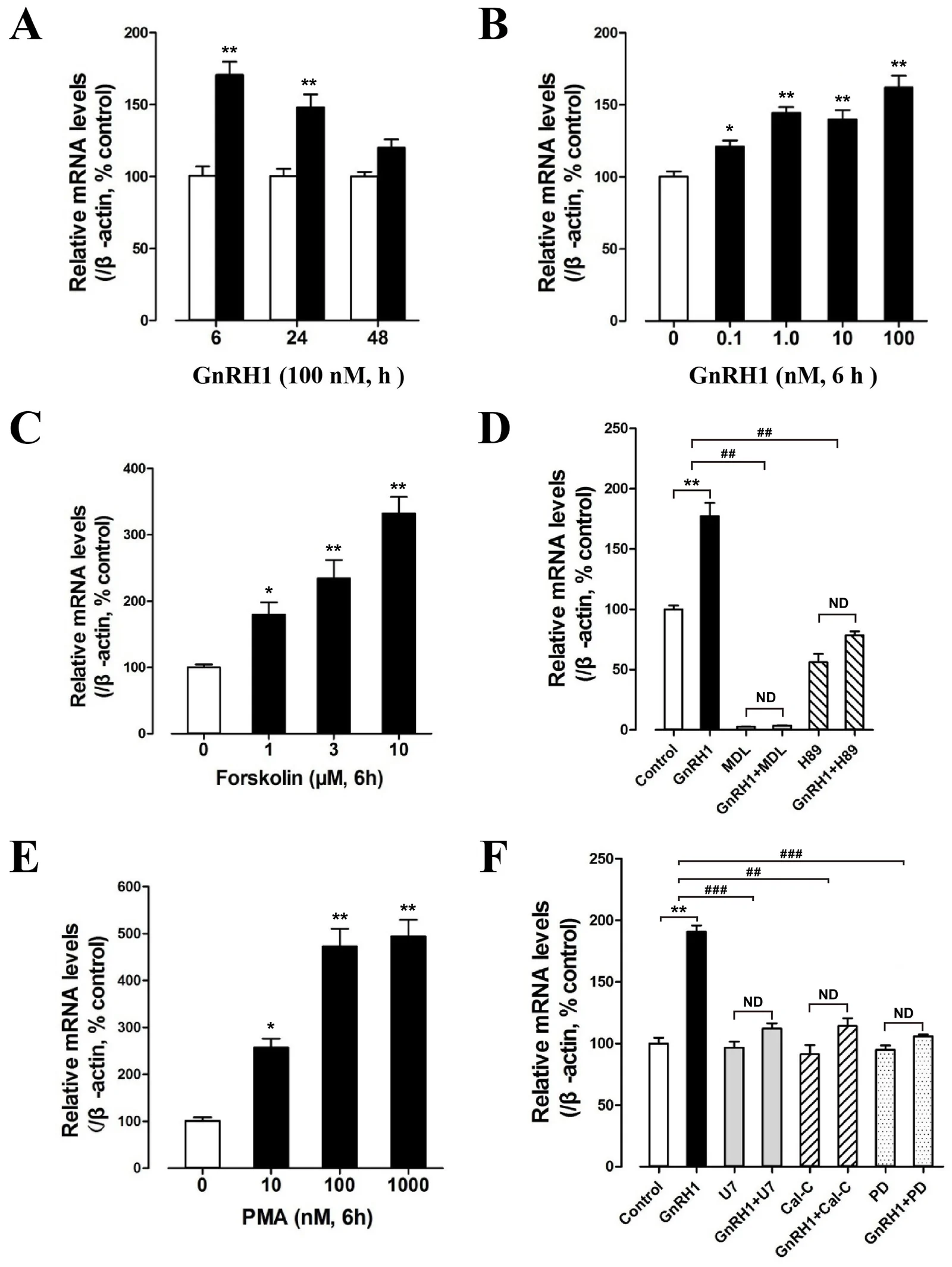
Regulation of chicken *GRP* expression by GnRH1 in cultured pituitary cells. **(A)** GnRH1 increased pituitary *GRP* mRNA abundance in a time-dependent manner. Chicken pituitary cells were incubated with GnRH1 for various duration (6h, 24h or 48h, respectively). **(B)** GnRH1 increased pituitary *GRP* mRNA abundance in a dose-dependent manner. Chicken pituitary cells were incubated with various concentrations of GnRH1. **(C)** Effects of forskolin on basal *GRP* mRNA expression. Chicken pituitary cells were incubated with various concentrations of forskolin. **(D)** Effects of MDL-12,330A and H89 on GnRH1-induced *GRP* mRNA expression. Chicken pituitary cells were treated with chicken GnRH1 (10 nM) for 6 h, in the presence or absence of pharmacological agents (MDL:20 μM, H89:20μM). **(E)** Effects of PMA on basal *GRP* mRNA expression. Chicken pituitary cells were incubated with various concentrations of PMA. **(F)** Effects of U73122 (U7), calphostin C (Cal-C), and PD98059 (PD) on GnRH1-induced *GRP* mRNA expression. Chicken pituitary cells were treated with chicken GnRH1(10 nM) for 6 h, in the presence or absence of pharmacological agents (U73122:20 μM, calphostin C: 200 nM, PD98059: 100 μM). Unless indicated otherwise, values are means ± SEM from four biological replicates (N = 4). ND indicates no significant difference between the indicated groups. *P < 0.01; **P < 0.001 *versus* the corresponding control. ^##^P < 0.01; ^###^P < 0.001 *versus* GnRH1 alone.

We next examined the signaling pathways involved in GnRH1-mediated regulation of *GRP* mRNA expression. Forskolin mimicked the stimulatory effect of GnRH1 and increased *GRP* expression in a dose-dependent manner (1-10 μM for 6 h; **Figure 2C**). The adenylyl cyclase (AC) inhibitor MDL-12,330A (20 μM) completely abolished the stimulatory effect of GnRH1 (10 nM for 6 h) on *GRP* expression and significantly reduced basal *GRP* expression (**Figure 2D**). Similarly, the protein kinase A (PKA) inhibitor H89 (20 μM) markedly suppressed the GnRH1 response and significantly down-regulated basal *GRP* expression (**Figure 2D**). These results suggests that GnRH1 likely regulate *GRP* mRNA expression through the AC/cAMP/PKA signaling pathway.

GnRH1 can also activate the PLC/PKC/MEK/ERK pathway through GnRH receptors (Millar, 2005). We therefore used PMA (a PKC activator), U73122 [a phospholipase C (PLC) inhibitor], calphostin C (a PKC inhibitor), and PD98059 (a MEK inhibitor) to examine their potential involvement in the regulation of pituitary *GRP* expression. PMA (10–1000 nM, 6h) mimicked the effect of GnRH1 and increased *GRP* mRNA expression in a dose-dependent manner (**Figure 2E**). GnRH1-induced (10 nM for 6 h) *GRP* expression was significantly inhibited by U73122 (U7, 20 μM), calphostin C (Cal-C, 200 nM), and PD98059 (PD, 100 μM) in cultured chicken pituitary cells (**Figure 2F**). These findings hint the potential involvement of the PLC/PKC/MEK/ERK signaling cascade in GnRH1-induced *GRP* expression.

### Regulation of *GRP* expression by sex steroids in cultured chicken pituitary cells

Pituitary hormone expression is often feedback regulated by sex steroids (Scanes, 2022a). We therefore examined the effect of DHT on chicken *GRP* expression. As shown in **Figure 3A**, DHT (1–100 nM for 48 h) down-regulated *GRP* mRNA abundance in cultured pituitary cells in a dose-dependent manner, suggesting DHT downregulates pituitary *GRP* expression.

**Figure 3:**
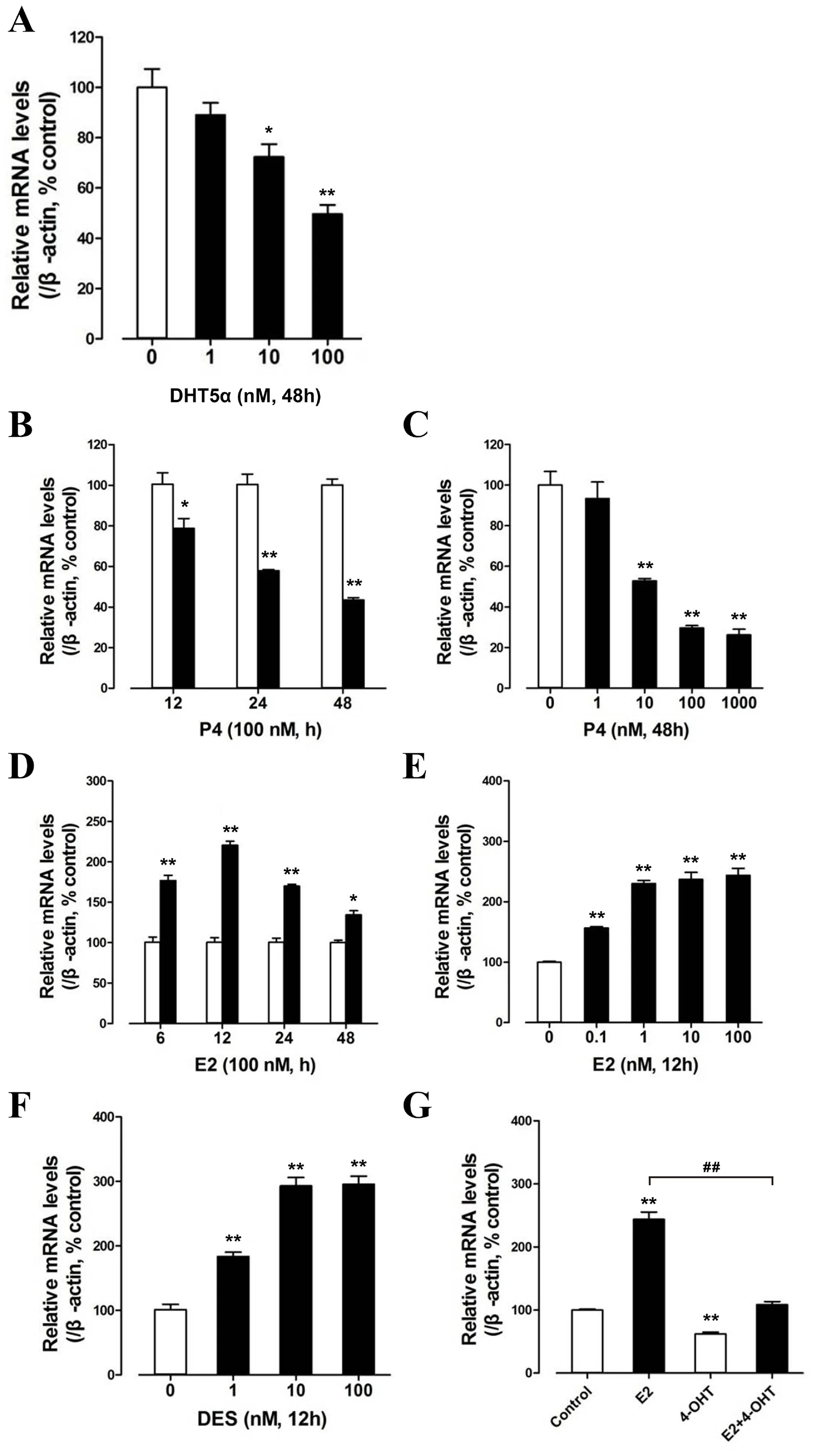
Regulation of chicken *GRP* expression by sex steroids in cultured pituitary cells. **(A)** DHT5α down-regulated chicken *GRP* expression in a dose-dependent manner. Chicken pituitary cells were incubated with various concentrations of DHT5αfor 48 h. **(B)** P4 down-regulated the chicken *GRP* expression in a time-dependent manner. Chicken pituitary cells were incubated with P4 for various duration. **(C)** P4 down-regulated the chicken *GRP* expression in a dose-dependent manner. Chicken pituitary cells were incubated with P4 at various concentrations. **(D)** E2 up-regulated the chicken *GRP* expression. Chicken pituitary cells were incubated with E2 for various duration (6h, 12h, 24h or 48h, respectively). **(E)** E2 up-regulated the chicken *GRP* expression in a dose-dependent manner. Chicken pituitary cells were incubated with E2 at various concentrations. **(F)** Dose-dependent effect of DES on *GRP* mRNA expression. Chicken pituitary cells were incubated with DES at various concentrations. **(G)** Effect of 4-OHT on E2-induced *GRP* mRNA expression. Chicken pituitary cells were treated with E2 (1 nM) for 12 h, in the presence or absence of 4-OHT (1 μM). Values are means ± SEM from four biological replicates (N = 4). *P < 0.05; **P < 0.001 *versus* the corresponding control; ^##^*P* < 0.001 *versus* E2 treatment without 4-OHT.

Progesterone (P4) is a steroid hormone secreted by granulosa cells of growing ovarian follicles (Kato et al., 1995; Caicedo Rivas, 2004). We also examined its effect on *GRP* expression. As shown in **Figures 3B, C**, P4 down-regulated *GRP* mRNA abundance in both time-(100 nM, 12–48 h) and dose-dependent manners (1–1000 nM, 48 h).

17β-Estradiol (E2) is a major sex steroid involved in the development and regulation of the female reproductive system and secondary sexual characteristics (Scanes, 2022b). To determine whether E2 regulates *GRP* expression, we treated the cultured chicken pituitary cells with E2 and measured *GRP* mRNA level by qPCR. E2 up-regulated *GRP* expression in a time-dependent manner (100 nM, 6–48 h), with the maximal response observed after 12 h (**Figure 3D**). E2 also increased *GRP* expression in a dose-dependent manner (0.1–100 nM, 12 h), with a minimum effective concentration of 0.1 nM (**Figure 3E**). DES, an E2 analogue, similarly induced *GRP* expression in a dose-dependent manner after 12 h treatment (**Figure 3F**). By contrast, 4-hydroxytamoxifen (4-OHT), an estrogen receptor antagonist, markedly inhibited the effect of E2 (**Figure 3G**), suggesting that E2 possibly regulates *GRP* expression through estrogen receptors.

### Sequence and promoter activity of the 5^′^-flanking region of the chicken *GRP* gene

The 5′-flanking region of the chicken *GRP* gene was cloned using sequence information from the chicken genome database (*Gallus gallus*, genome assembly bGalGal1.mat.broiler.GRCg7b; INSDC accession GCA_016699485.1). The amplified region was 1,914 bp long and showed a GC content of 57.26%. Putative transcription factor-binding sites within 915-bp upstream of the transcription start site were predicted using JASPAR-A database of transcription factor binding profiles and BDGP: Neural Network Promoter Prediction (**Figure 4**). Two putative Sp1-binding sites (GC boxes) were predicted near the transcription start site. Putative binding sites were also identified for the transcription factors: NFAT5, HOXA13, PRDM1, GATA3, ZIC2, ZEB1, FOSL1, KLF9, KLF2, SMAD2, E2F, E2F4, WT1, KLF3, E2F6, and PAX6. Notably, two putative estrogen receptor binding sites, designated estrogen receptor 1 binding site (ESR1) and estrogen receptor 2 binding site (ESR2), were located at positions -914 and -411 bp, respectively.

**Figure 4:**
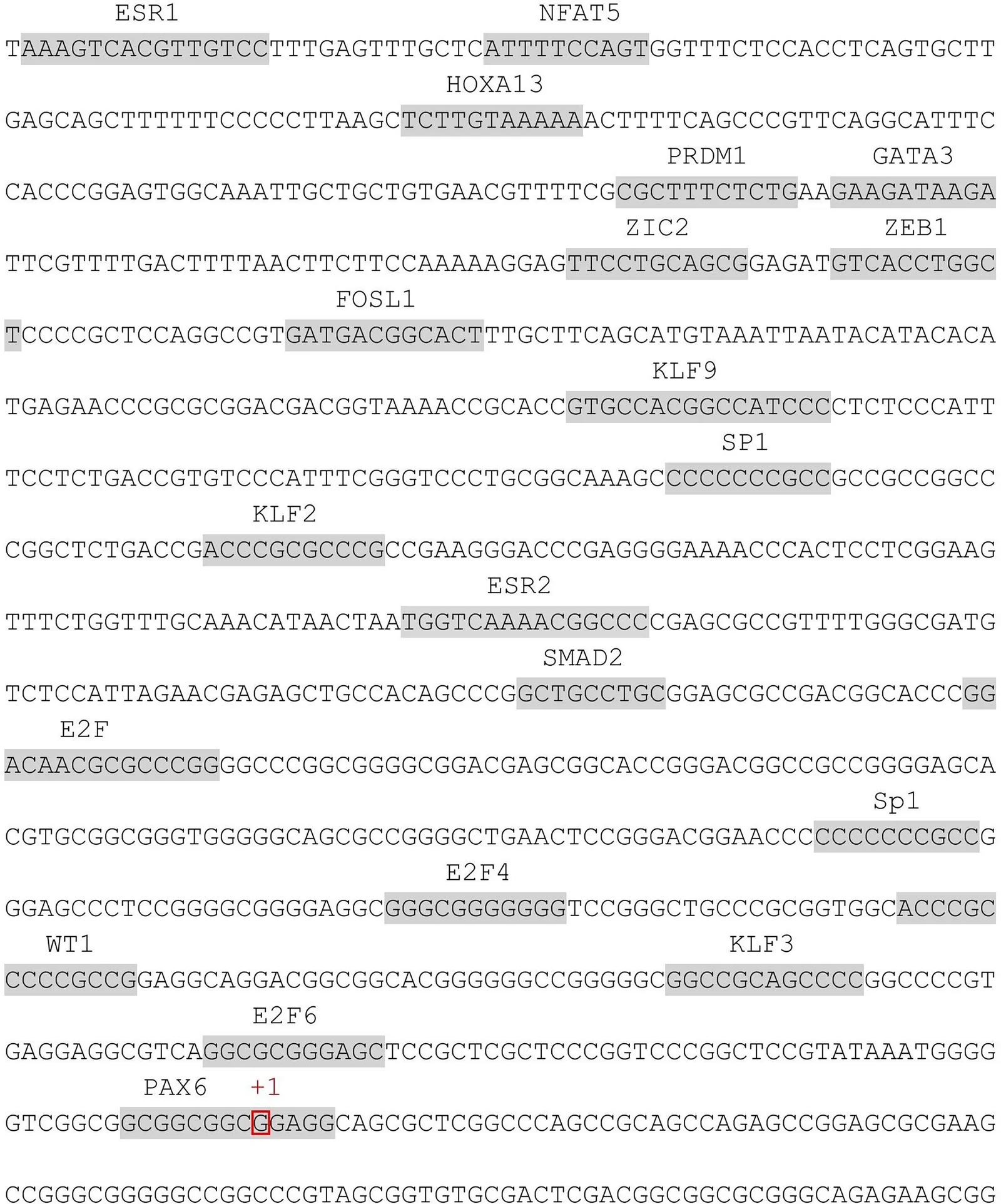
Nucleotide sequence of the chicken *GRP* 5′-flanking region extending 915 bp upstream of the transcription start site. Predicted transcription factor-binding sites are shaded and include sites for NFAT5, HOXA13, PRDM1, GATA3, ZIC2, ZEB1, FOSL1, KLF9, KLF2, SMAD2, E2F, E2F4, WT1, KLF3, E2F6, and PAX6. The nucleotide G at the transcription start site is enclosed in a red box and designated +1.

To determine whether the chicken *GRP* 5′-flanking region exhibited promoter activity, we subcloned the region into the promoter-less pGL3-Basic vector and examined luciferase activity in DF-1 and HEK293 cells. The 1,914-bp fragment exhibited strong promoter activity in both cell lines (**Figure 5**). Activity was higher when transfected in DF-1 cells (approximately 16-fold above the pGL3-Basic control; **Figure 5B**) and was lower in HEK293 cells (approximately 12-fold above the pGL3-Basic control; **Figure 5A**). Deletion of the distal region from -1,914 to -914 bp did not significantly reduce promoter activity in either cell line, indicating that basal promoter activity resides primarily within the proximal -915/+86-bp region.

**Figure 5:**
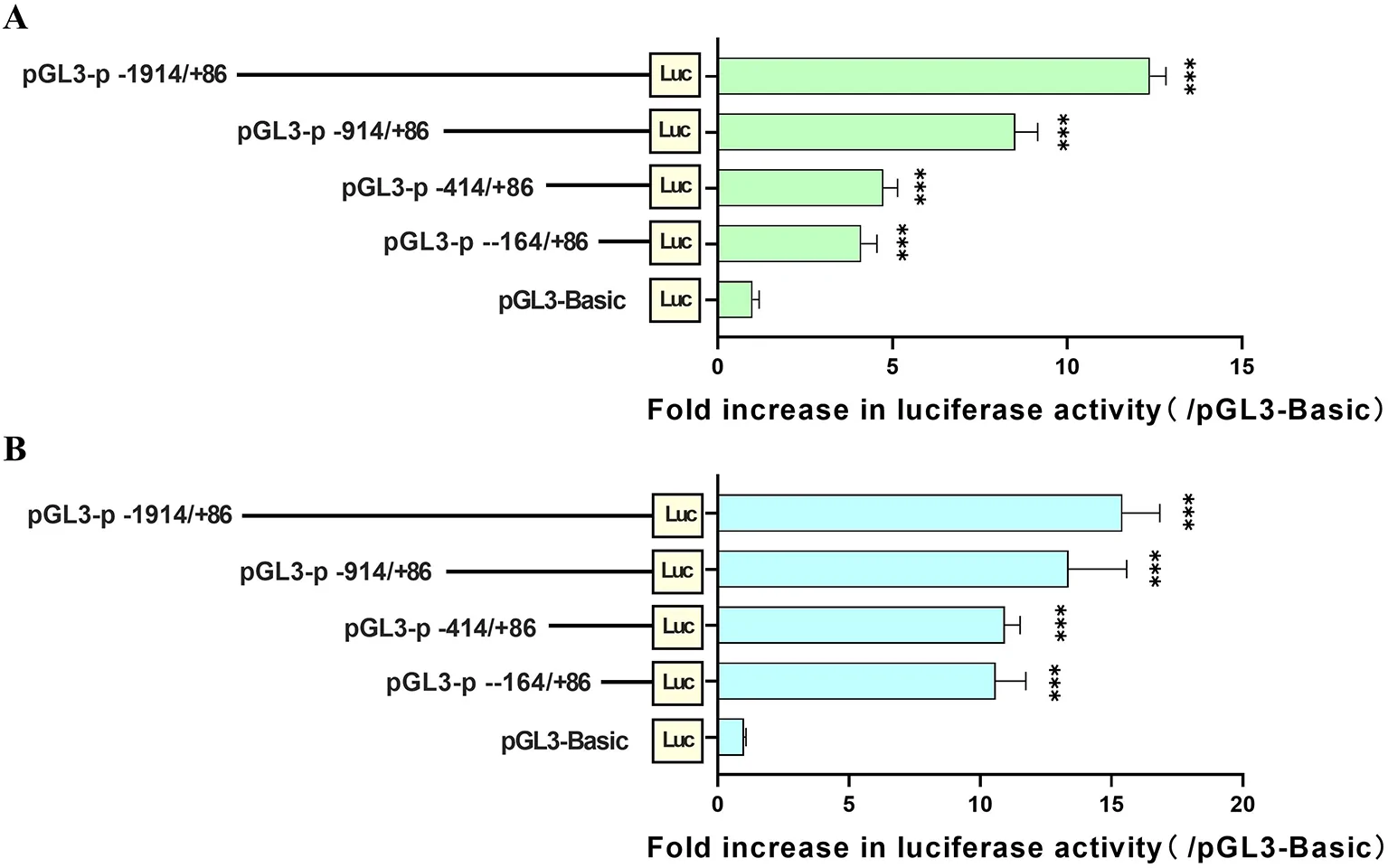
Promoter activity of the chicken GRP 5′-flanking region in human embryonic kidney (HEK293) cells **(A)** and chicken embryo fibroblast (DF-1) cells **(B)**. The p(-1914/+86)-Luc, p(-914/+86)-Luc, p(-414/+86)-Luc, and p(-164/+86)-Luc constructs were co-transfected with pRL-TK. The pRL-TK vector was used to normalize transfection efficiency, and a promoter-less pGL3-Basic vector was included as a control. Relative promoter activity is expressed as the fold change over the corresponding pGL3-Basic control, whose activity was set to 1, after normalization to pRL-TK activity. Values are means ± SEM from three independently transfected wells in one representative experiment; the experiment was independently repeated at least twice. Data were analyzed by one-way ANOVA followed by Dunnett’s multiple-comparisons test. ***P < 0.001 versus the corresponding pGL3-Basic control.

### Effects of E2 on chicken *GRP* promoter activity and identification of potential estrogen receptor-binding sites

Two putative estrogen receptor binding sites, designated ESR1 and ESR2, were predicted within the chicken *GRP* 5’-flanking region, suggesting its potential regulation by E2. The effect of E2 on *GRP* promoter activity in DF-1 cells was examined in this study. E2 significantly induced *GRP* promoter activity (**Figure 6A**), revealing its regulation possibly through binding to GRP promoter region.

**Figure 6:**
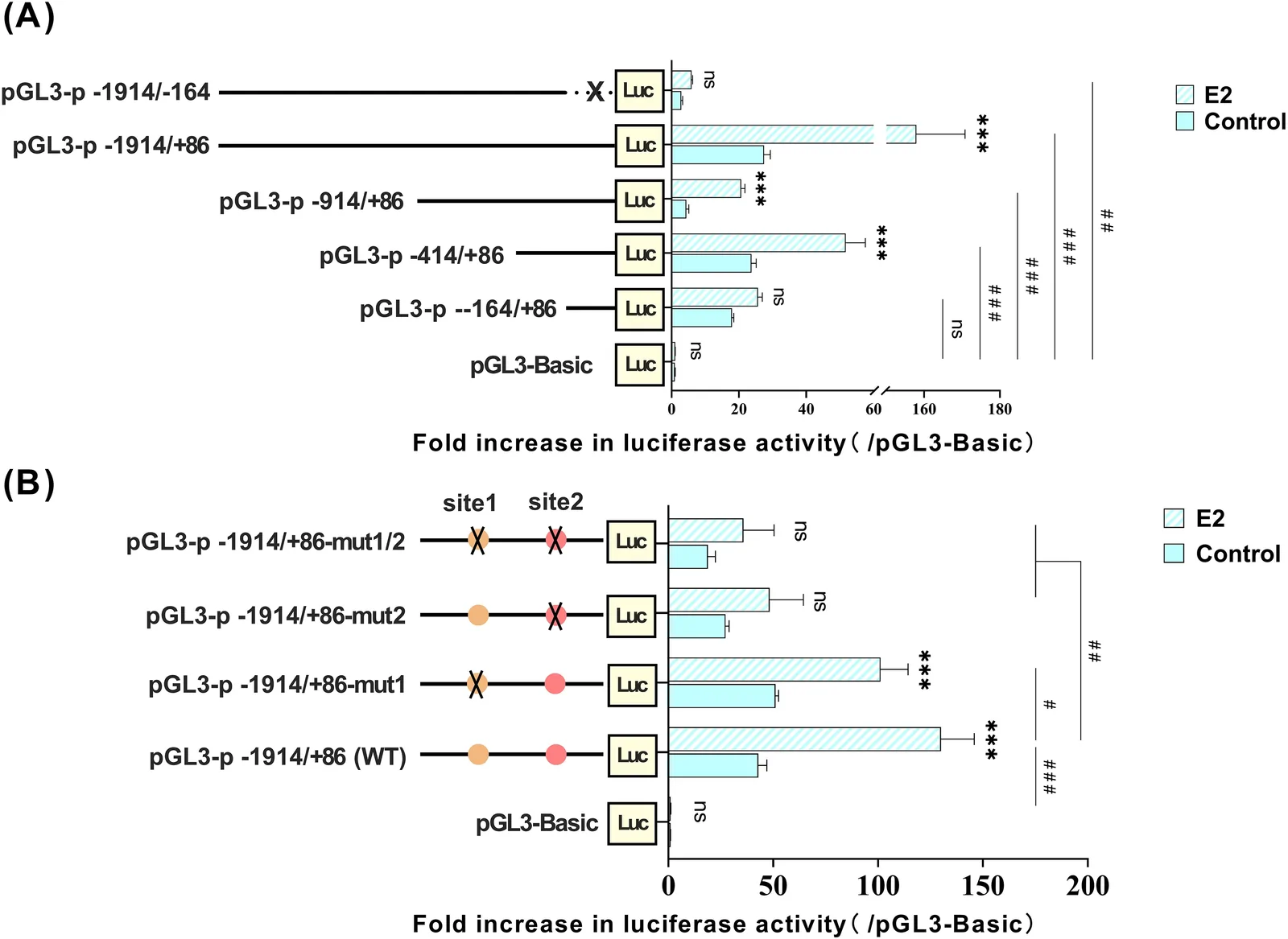
Effects of E2 on chicken *GRP* promoter activity and identification of potential estrogen receptorbinding sites. **(A)** The p(-1914/+86)-Luc, p(-914/+86)-Luc, p(-414/+86)-Luc, and p(-164/+86)-Luc constructs were co-transfected into DF-1 cells with pcDNA3.1-ESR1, pcDNA3.1-ESR2, and pRL-TK. At 24 h after transfection, cells were treated with 100 nM 17β-estradiol (E2). **(B)** The p(-1914/+86)-Luc(WT), p(-1914/+86)mut1-Luc, p(-1914/+86)mut2-Luc, and p(-1914/+86)mut1/2-Luc constructs were co-transfected into DF-1 cells with pcDNA3.1-ESR1, pcDNA3.1-ESR2, and pRL-TK, followed by treatment with E2 (100 nM) at 24 h after transfection. In both experiments, pRL-TK was used to normalize transfection efficiency, and a promoter-less pGL3-Basic vector was included as a control. Relative promoter activity is expressed as the fold change over the pGL3-Basic control, whose activity was set to 1, after normalization to pRL-TK activity. Values are means ± SEM from three biological replicates (N = 3, representing three independently transfected wells in the representative experiment shown). Data were analyzed by two-way ANOVA followed by Bonferroni’s *post hoc* test. ***P < 0.001 for E2 *versus* control within each construct; ^#^*P* < 0.05; ^##^*P* < 0.01; ^###^*P* < 0.001 *versus* WT under the same treatment condition. ns, not significant.

To determine whether the two putative estrogen receptor binding sites are involved for E2-induced GRP promoter activity, three site-directed deletion constructs were employed in present study. As shown in **Figure 6B**, E2 significantly increased the activity of the WT and mut1 constructs, whereas no significant E2 response was detected for the mut2 or mut1/2 constructs. The additional between-constructs analyses showed that the E2 responses of mut2 or mut1/2 was significantly reduced relative to that of the WT construct. These results indicate that both sites were potential for the E2-dependent binding.

### Differential regulation of *GRP* and *FSHβ* expression by E2 in cultured chicken pituitary cells

Follicle-stimulating hormone (FSH) is secreted by pituitary gonadotrophs and mediates multiple reproductive processes (Scanes, 2022b). GRP is also abundantly expressed in chicken gonadotrophs and promotes the survival of small ovarian follicles (Mo et al., 2017, 2025). We therefore compared the effects of E2 on *FSHβ* and *GRP* expression in cultured chicken pituitary cells. E2 down-regulated *FSHβ* mRNA abundance but increased *GRP* mRNA abundance (**Figure 7**).

**Figure 7:**
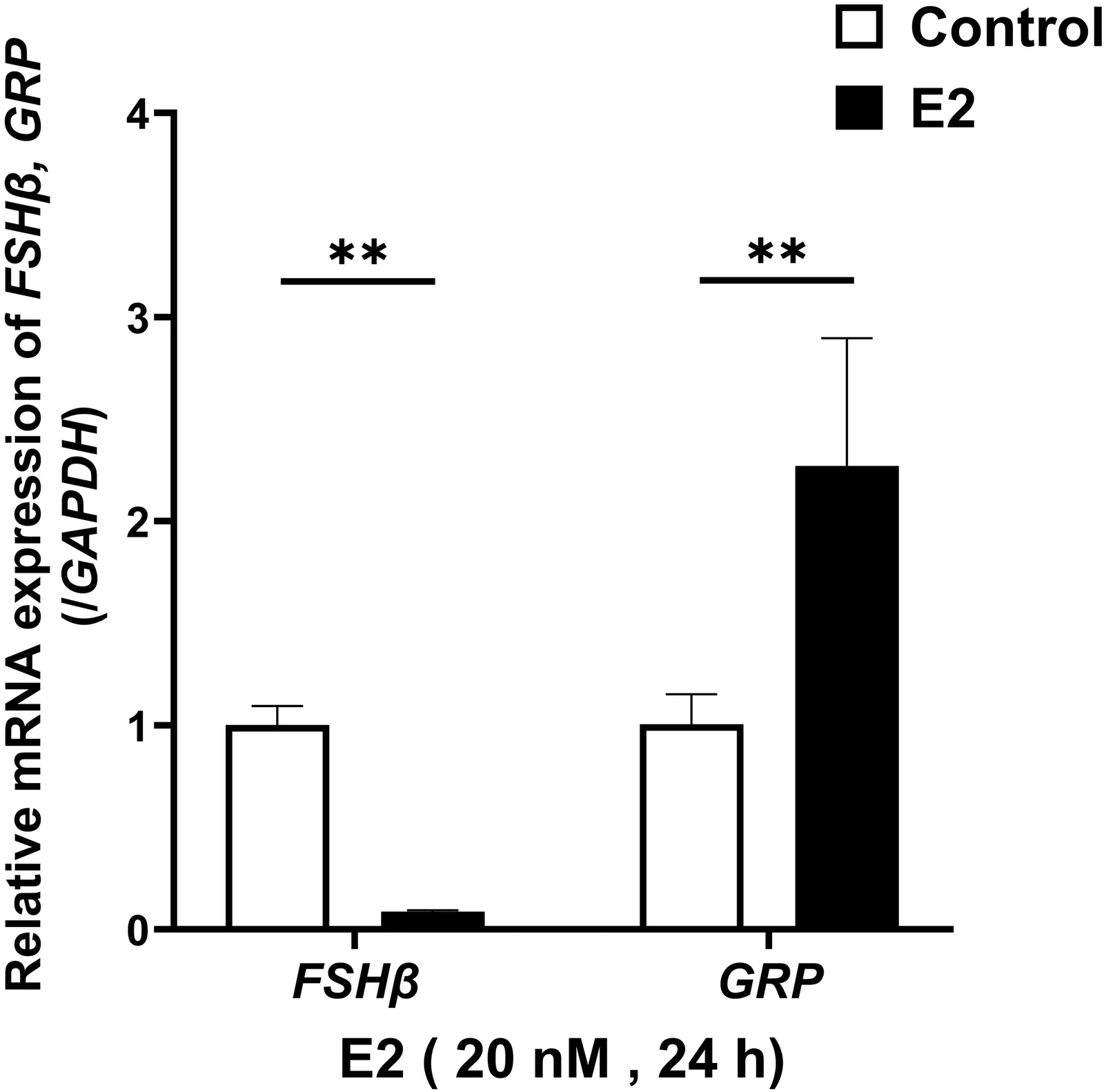
Differential regulation of pituitary *FSHβ* and *GRP* expression by E2. Chicken pituitary cells were incubated with E2 (20nM) for 24h, after which *FSHβ* and *GRP* expression was measured by qPCR. Values are means ± SEM from four biological replicates (*N= 4*). **P < 0.01 *versus* the corresponding control.

## Discussion

This study shows for the first time that GnRH1 regulates *cGRP* expression in cultured chicken pituitary cells. The sex steroids, DHT and P4, down-regulat *GRP* expression, whereas E2 up-regulats *GRP* expression in dose- and time-dependent manners. Promoter analysis further identified potential estrogen receptor-binding sites, which provides a molecular basis for E2 up-regulation of *cGRP* in cultured pituitary cells. Together with the reported role of GRP in the survival of small ovarian follicles, these findings suggests that GRP plays a role in the regulation of egg production in chickens.

Analysis of the single-cell atlas showed that *GRP* is particularly abundant in female gonadotrophs. This finding is consistent with our previous observation that GRP-immunoreactive cells primarily colocalize with LH-immunoreactive cells (Mo et al., 2017). The distribution of GRP resembles that of the putative chicken pituitary hormone RLN3, which is also abundant in adult female gonadotrophs (Lv et al., 2022), but differs from that of the putative pituitary hormone CART, which shows no clear gender bias (Mo et al., 2015). As gonadotrophs can be further divided into four subpopulations, GRP may be enriched in a subpopulation characterized by high *LHβ* and *RLN3* expression (Leng et al., 2024).

GnRH1 regulated *cGRP* mRNA expression in both dose- and time-dependent manners, consistent with the central role of GnRH1 in pituitary reproductive hormone synthesis and secretion (Seeburg et al., 1987). GnRH1 is processed from a precursor polypeptide in hypothalamic neurons, and GnRH-containing granules are transported along axons to the external zone of the median eminence (Okubo and Nagahama, 2008). In Japanese quails, FSHβ synthesis and secretion are regulated by GnRH both *in vitro* and *in vivo* (Hattori et al., 1985, 1986). Similar findings (Hattori et al., 1985, 1986) have been reported where LH synthesis and secretion are tightly controlled by GnRH1 (Sharp et al., 1990). Since low-frequency pulsatile GnRH favors *FSHβ* transcription whereas its high-frequency stimulation favors *LHβ* transcription (Papavasiliou et al., 1986; Turgeon et al., 1996), the effects of GnRH1 on GRP expression and secretion warrant further investigation *in vitro* and *in vivo*.

GnRH1 signals through its receptors GnRHR1 and GnRHR2 (Fanis et al., 2023). Given the abundant expression of GnRHR2 in the chicken pituitary (**Figure 1F**) and their signaling pathways revelation (Joseph et al., 2009), our pharmacological data suggests that GnRH1 induces *GRP* expression likely via the AC/cAMP/PKA pathway. Forskolin, an adenylyl cyclase (AC) activator, increases the intracellular cAMP and induced *GRP* expression, whereas the AC inhibitor MDL-12330A and the PKA inhibitor H89 blocked the effect of GnRH1. These findings are consistent with evidence that activated chicken GnRHR2 can stimulate the cAMP/PKA signaling in GH3 cells (Shimizu and Bédécarrats, 2010). GnRH1-mediated regulation of *GRP* expression through GnRHR2 may therefore resemble its regulation of *FSHβ* expression in LβT2 cells, where low-frequency pulsatile GnRH preferentially promotes *FSHβ* expression (Turgeon et al., 1996).

Our results also indicate that the PLC/PKC/MEK/ERK pathway may be involved in GnRH1-induced *GRP* expression. PMA, a potent PKC activator, mimicked GnRH1 in up-regulation of *GRP* mRNA expression in a dose-dependent manner (**Figure 2E**). In addition, U73122, calphostin C, and PD98059 inhibited GnRH1-induced *GRP* expression in cultured chicken pituitary cells. These observations are consistent with evidence that activated GnRHR2 increases intracellular IP3 in COS-7 cells (Joseph et al., 2009).The involvement of the PLC/PKC/MEK/ERK signaling in *GRP* expression regulation also parallels the CART synthesis control (Mo et al., 2019). In addition, depletion of extracellular calcium or treatment with calcium-channel blockers reduces GnRH-induced LH release from chicken pituitary cells (Davidson et al., 1988; Liu et al., 1995), whereas artificial activation of the PLC pathway increases LH release (Johnson and Tilly, 1991).

5α-dihydrotestosterone (DHT) is an androgen that contributes to the development and maintenance of male characteristics. In this study, DHT inhibited *GRP* expression in cultured chicken pituitary cells. This response differs from that observed in rats, in which DHT positively regulates *GRP* expression (Oti et al., 2018). Oti et al. reported that Venus fluorescence driven by a GRP promoter construct in the male lumbar spinal cord decreased markedly after castration and was restored by chronic androgen replacement. The contrasting responses to DHT suggest species- and tissue-specific regulation of *GRP* expression (Oti et al., 2018).

Progesterone (P4) is a sex steroid that prepares the uterine endometrium for implantation (Cope and Monsivais, 2022) and supports the uterine environment during pregnancy (Raperport et al., 2023). In cultured chicken pituitary cells, P4 down-regulated *GRP* expression in both dose- and time-dependent manners. This finding is consistent with observations in a GRP-promoter-Venus transgenic rat line (Oti et al., 2018), in which chronic progesterone treatment attenuated androgen-induced Venus expression in the spinal cord of adult males (Oti et al., 2018). By contrast, progesterone did not alter *GRP* expression in cultured pituitary cells from ovariectomized sheep (Whitley et al., 2000), substantiating potential species- or tissue-dependent regulation.

E2 up-regulated *GRP* expression in both time- and dose-dependent manners. This response differs from that observed in sheep, in which estrogen did not affect *GRP* expression in cultured pituitary cells (Whitley et al., 2000). Our promoter analysis mapped the basal *GRP* promoter activity primarily to the region proximal to the transcription start site (-915/+86 bp). Deletion of the putative estrogen receptor-binding sites reduced promoter activity *in vitro*, whereas E2 increased promoter activity when these sites were intact. These findings provide a molecular basis for E2-mediated up-regulation of *GRP* expression. They may also have implications for regulation of the GRP/GRPR system in hormone-responsive conditions. E2 has been reported to increase female itch sensitivity by upregulating spinal neurons that express GRPR (Takanami et al., 2021). In addition, E2 downregulates E-cadherin in breast cancer, in which the GRP/GRPR system can be highly expressed (Raymond et al., 2025). The present findings raise the possibility that E2-mediated regulation of GRP contributes to tumor growth or metastasis, although this hypothesis requires further elucidation.

Follicular development is tightly regulated by the pituitary gonadotropins, FSH and LH, which collectively promote follicular growth, maturation, and ovulation (Vitale and Dolmans, 2024; Lai et al., 2026). E2 down-regulated *FSHβ* expression (**Figure 7**), which is consistent with the findings in birds (Kirby et al., 2005), sheep (Phillips et al., 1988), humans (Marshall et al., 1983; Welt et al., 2003), and fish (Mizrahi et al., 2025), mainly be involved in the post-hierarchal follicles growth and steroid synthesis (Uehara et al., 2024). By contrast, the E2 up-regulated GRP expression which is first reported in present study, may be mainly keep the pre-hierarchal follicles survival (Mo et al., 2025). Their differential regulation profiles by E2, in combination with their receptors differentially expressed along the pre- and post- hierarchal follicles (Zhang et al., 1997), support the characteristic pituitary-ovary interaction in avian species.

In present study, the E2-mediated increase in *GRP* expression suggests that this putative pituitary hormone may be involved in the regulation of egg production. cGRP activates the intracellular MAPK/ERK signaling (Mo et al., 2017) and promotes granulosa cell proliferation. Because *cGRPR* is abundant in granulosa cells of small ovarian follicles (Mo et al., 2025), cGRP may promote the survival of the large pool of small follicles. E2-mediated upregulation of *GRP* may therefore participate in a positive feedback loop in which growing follicles produce more E2, and an enhanced E2 level stimulates higher pituitary *GRP* expression, thus promoting the survival of more small follicles with the potential to enter the preovulatory hierarchy (**Figure 8**). This positive feedback loop may also provide a mechanistic context for the association between circulating steroid concentrations and egg production reported by Leszczynski et al (Leszczynski et al., 1985). In hens from eight genetic stocks that differed in age and egg production, the basal circulating E2/P4 ratio was a more informative estimator of egg production than either hormone alone. According to the three-cell model of ovarian E2 synthesis, P4 is first synthesized by granulosa cells, converted to androgen by theca interna cells, and then converted to E2 by theca externa cells (Kato et al., 1995) (London and Clayton, 2010). The association of E2/P4 ratio with egg production reflects coordination between granulosa and theca cells (Jozkowiak et al., 2020; Li et al., 2022). Adequate P4 production may indicate a large population of proliferatively active granulosa cells, a process that may be supported by GRP.

**Figure 8:**
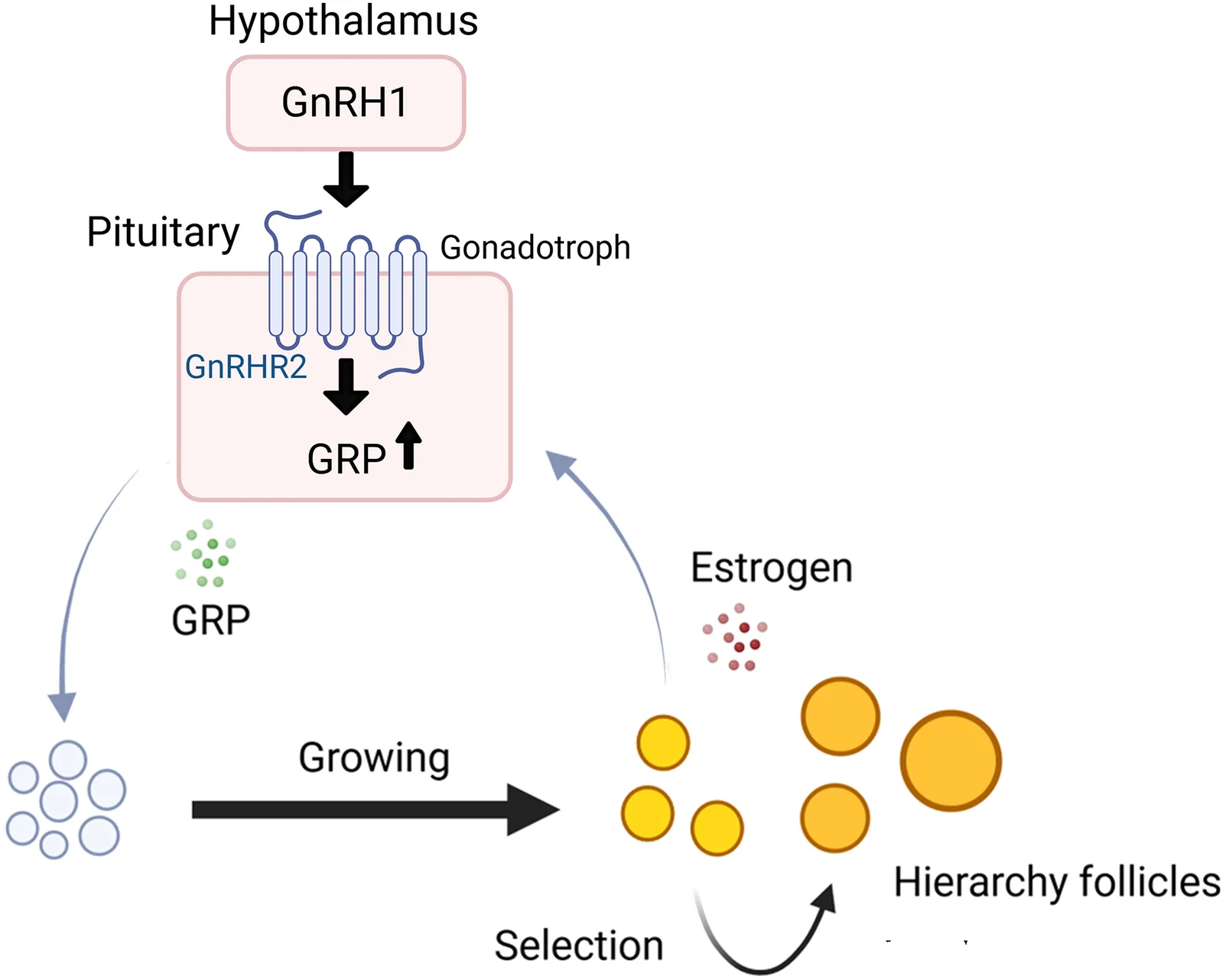
Hypothesis model illustrating the effect of GRP on follicular growth.

In conclusion, this study shows for the first time that GnRH1 regulates *cGRP* expression, supporting a central role for GnRH1 signaling in the control of reproductive hormone synthesis. The sex steroids, DHT and P4, down-regulates *GRP* expression in cultured chicken pituitary cells, whereas E2 up-regulates *GRP* expression possibly through the two potential estrogen receptors binding sites within the 5’flanking region of *GRP* gene. Given the mitogenic effect of GRP on granulosa cells and its reported role in follicular survival, these findings suggest GRP plays a role in the regulation of egg production in chickens.

This study has some limitations. First, pharmacological studies suggest the possible involvement of the many factors involved in signaling pathways. Direct measurement of intracellular cAMP, PKA activation, PKC activation, or intracellular calcium level will help validate the signaling mechanisms behind GnRH1-regulated GRP expression. Second, besides transcriptional regulation via direct binding to the potential estrogen receptor-binding sites, the effect of E2 on GRP transcription activity may also be affected by the distal spacing, chromatin-independent transcription factor interactions, or other sequence elements. Hence, receptor knockdown or receptor-specific loss-of-function experiments may provide further elucidation. Third, GRP secretion may vary across chicken breeds or be limited in some breeds, thus the effect of GRP on follicular growth remains a hypothesis that requires further testing, for instance, gene knockout studies.

## Data Availability

The datasets presented in this study can be found in online repositories. The names of the repository/repositories and accession number(s) can be found below: https://ngdc.cncb.ac.cn/gsa/, CRA003604.
